# Modulation of the Expression of the Proinflammatory IL-8 Gene in Cystic Fibrosis Cells by Extracts Deriving from Olive Mill Waste Water

**DOI:** 10.1155/2013/960603

**Published:** 2013-07-07

**Authors:** Ilaria Lampronti, Monica Borgatti, Silvia Vertuani, Stefano Manfredini, Roberto Gambari

**Affiliations:** ^1^Department of Life Science and Biotechnology, Laboratory of Medicinal and Molecular Biotechnology, University of Ferrara, Via Fossato di Mortara 74, 44121 Ferrara, Italy; ^2^Health Products Laboratory, University of Ferrara, Via Fossato di Mortara 17/19, 44121 Ferrara, Italy

## Abstract

A persistent recruitment of neutrophils in the bronchi of cystic fibrosis (CF) patients contributes to aggravate the airway tissue damage, suggesting the importance of modulating the expression of chemokines, including IL-8 during the management of the CF patients. Polyphenols rich extracts derived from waste water from olive mill, obtained by a molecular imprinting approach, have been investigated in order to discover compounds able to reduce IL-8 expression in human bronchial epithelial cells (IB3-1 cells), derived from a CF patient with a ΔF508/W1282X mutant genotype and stimulated with TNF-alpha. Initially, electrophoretic mobility shift assays (EMSAs) were performed to determine whether the different active principles were able to inhibit the binding between transcription factor (TF) NF-kappaB and DNA consensus sequences. Among different representative active principles present in the extract, three compounds were selected, apigenin, oleuropein, and cyanidin chloride, which displayed remarkable activity in inhibiting NF-kappaB/DNA complexes. Utilizing TNF-alpha-treated IB3-1 cells as experimental model system, we demonstrated that apigenin and cyanidin chloride are able to modulate the expression of the NF-kappaB-regulated IL-8 gene, while oleuropein showed no effect in regulating the expression of the gene IL-8.

## 1. Introduction

Cystic fibrosis (CF) is a severe and diffuse recessive genetic disease due to defects of the CF transmembrane conductance regulator (CFTR) gene [1]. CF affects several organs, with the chronic pulmonary disease being the major cause of reduction of the quality and expectancy of life. The hallmark of CF lung disease is the chronic infection generally sustained by the gram-negative bacterium *Pseudomonas aeruginosa* and excessive lung inflammation with a huge infiltrate of neutrophils in the bronchial lumen, mainly due to the release of the chemokine interleukin-8 (IL-8) [2–5]. The identification of innovative drugs able to reduce the excessive lung inflammation in CF patients is considered a promising therapeutic target to prevent the progressive lung tissue deterioration.

We have a continuous interest in finding novel active compounds, capable of inhibiting the biological activity of the NF-kappaB transcription factor (NF-*κ*B TF) [6–8] and showing inhibitory activity of IL-8 transcription in bronchial epithelial cells exposed to *P. aeruginosa* or TNF-*α* (tumor necrosis factor-alpha). In order to identify novel molecules, we have focused our attention on natural plants extracts, as a source of potential regulators of proinflammatory genes. In particular, we were interested in studying the possible use in medicinal chemistry of by-products of food and agroindustry companies, in order to propose strategies aimed at the full usage of material(s) (including waste materials) employed in food production. The hypothesis we are following is that in waste materials, chemical products might be present exhibiting biological activities suitable for biomedical applications [9].

The aim of the present study is to determine the activity of components, present in a new extract deriving from olive mill waste water, on the expression of IL-8 gene, the major chemokine released from CF cells under the control of NF-kappaB. NF-kappaB is a transcription factor that plays a crucial role in cell cycle regulation, in the expression of specific genes, in the regulation of cell differentiation, and apoptosis. Alteration of NF-kappaB activity is associated with several human pathologies, including osteoporosis, rheumatoid arthritis, and cancer. In addition, NF-kappaB is a critical transcription factor responsible for inflammatory process in CF cells [10]. Therefore, targeting NF-kappaB appears to be a relevant therapeutic strategy, as recently described and demonstrated [11–13].

The olive extract was obtained through an extractive process, based on the molecular imprinting concept, able to concentrate glycosylated polyphenols. It is known that, in general, molecularly imprinted polymers (MIPs) have been widely utilized for the imprinting of different derivatives (pharmaceuticals, pesticides, carbohydrates, peptides, etc.) [14]. Chrysa Michailof and collaborators, for example, utilized, in their recently described studies [15], caffeic acid and p-hydroxybenzoic acid as templates, preparing two MIPs that were used for isolation of polyphenols from olive mill waste water samples without previous pretreatment.

**Figure 1:**
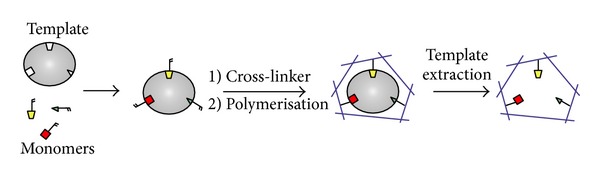
Principle of molecular imprinting: MIPs are prepared by allowing a network polymer to form, in presence of a template, a structure able to target analytes. Elimination of the template, after polymerization, leaves behind a cavity, which is complementary to the template in terms of size and shape. MIPs can be programmed to recognize a large variety of small molecules (*SciTopics*. Retrieved August 7, 2012, from http://www.scitopics.com/Molecular_Imprinting.html).

In our study, we utilized this extractive technique for its ability to separate specific compounds from complex mixtures. This procedure, termed by us Natural Molecular Imprinting technology (NMI), is based on molecular recognition, as well as in MIPs. However, the NMI takes advantage from the use of natural polymers obtained by a process of polymerization with monomers of chitin, and polyphenol glycosylated molecules as template (Figure 1).

**Figure 2:**
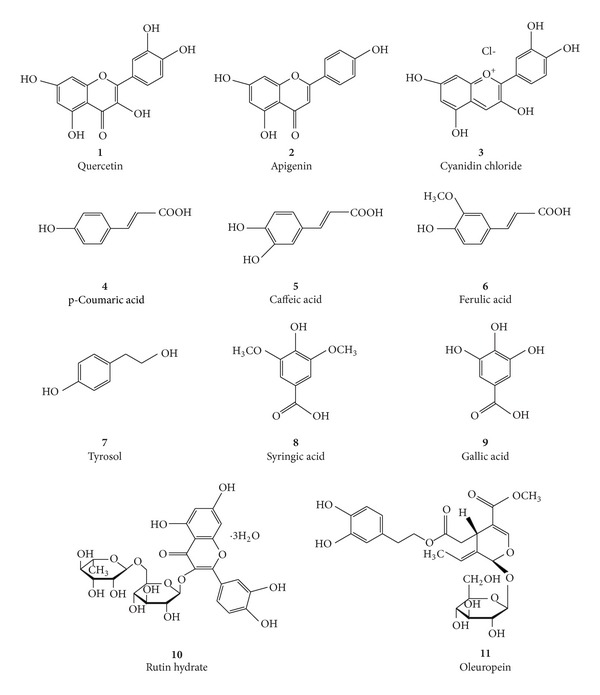
Chemical structures of active principles identified in olive oil extract.

The polymer matrix having cavities specific for this substrate was used as solid stationary phase during extraction on chromatographic column. With this method, we have obtained an extract rich in polyphenols, whose composition was determined by LC/MS analysis. The main components identified were oleuropein, verbascoside, salidroside, hydroxy-salidroside and its isomers, luteolin, rutin, apigenin, and anthocyanins (Figure 2).

**Figure 3:**
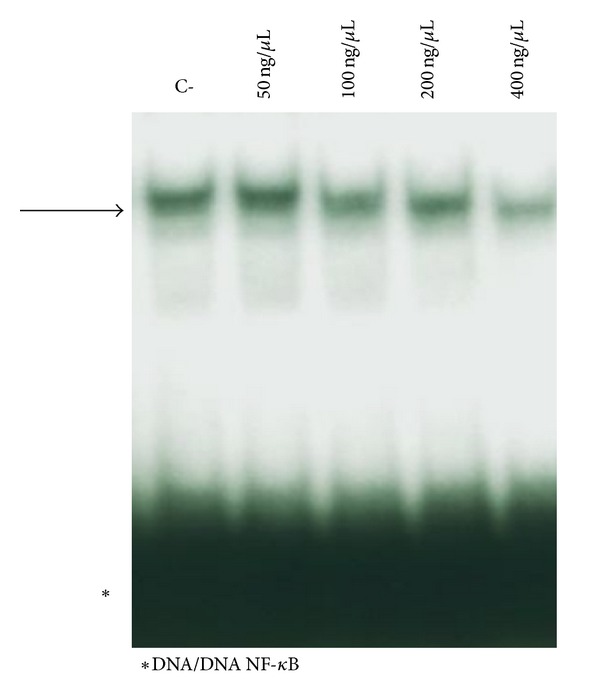
Effects of olive oil extract on the molecular interactions between NF-*κ*B nuclear extracts of K562 cells and ^32^P-labeled target NF-*κ*B double-stranded oligonucleotide. Olive extract was first incubated with NF-*κ*B, and then the ^32^P-labeled target NF-*κ*B oligonucleotide was added. NF-*κ*B/DNA complexes were analyzed by polyacrylamide gel electrophoresis. Arrows indicate NF-*κ*B/DNA complexes; asterisks indicate the free ^32^P-labeled target NF-*κ*B probe.

**Figure 4:**
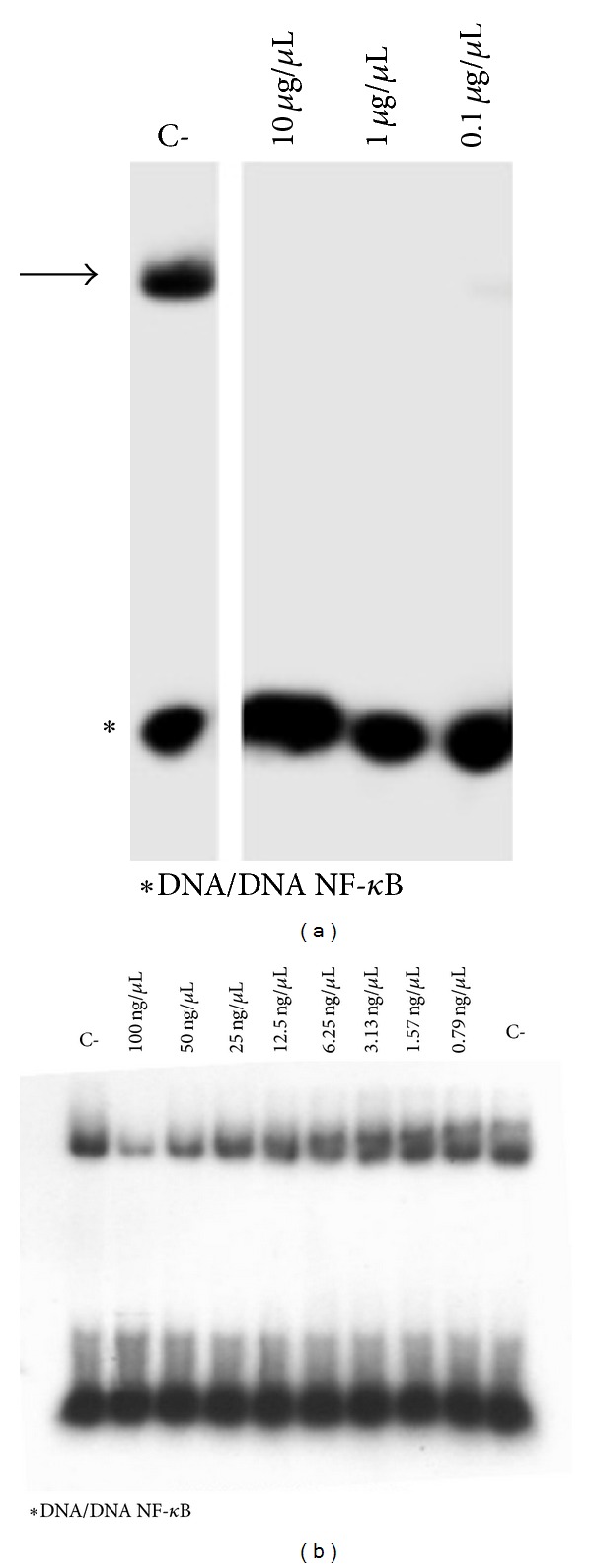
Effects of olive oil extract on the molecular interactions between NF-*κ*B p50 and ^32^P-labeled target NF-*κ*B double-stranded oligonucleotide. Extract was first incubated with NF-*κ*B, and then the ^32^P-labeled target NF-*κ*B oligonucleotide was added. NF-*κ*B/DNA complexes were analyzed by polyacrylamide gel electrophoresis. Arrows indicate NF-*κ*B/DNA complexes; asterisks indicate the free ^32^P-labeled target NF-*κ*B probe.

**Figure 5:**
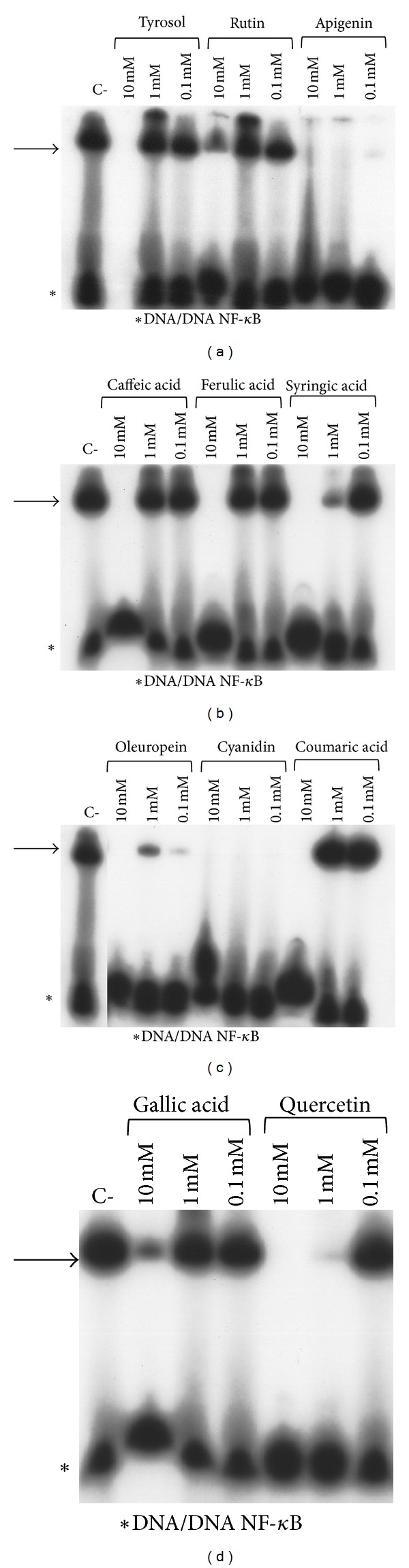
Effects of all the pure components identified in the olive oil extract on the molecular interactions between NF-*κ*B p50 and ^32^P-labeled target NF-*κ*B double-stranded oligonucleotide. Derivatives were first incubated with NF-*κ*B, and then the ^32^P-labeled target NF-*κ*B oligonucleotide was added. NF-*κ*B/DNA complexes were analyzed by polyacrylamide gel electrophoresis. Arrows indicate NF-*κ*B/DNA complexes; asterisks indicate the free ^32^P-labeled target NF-*κ*B probe.

The potential effect of the crude extract (Figures 3 and 4) and pure active principles (Figure 5) on the modulation of NF-kappaB interactions with target DNA sequences was investigated firstly by electrophoretic mobility shift assay (EMSA) experiments utilizing double-stranded ^32^P-labeled oligonucleotides as target DNA. Subsequently, TNF-*α* induced expression of IL-8 was evaluated by quantitative reverse transcription and polymerase chain reaction (Q-RT-PCR) in human bronchial epithelial cells IB3-1, derived from a CF patient with a ΔF-508/W1282X mutant genotype. This cellular system is very attractive, since it is well known that the hallmark in CF airway pathology is a characteristic elevated concentration of proinflammatory cytokines and chemokines, the most important of which seems to be IL-8 [16–18].

## 2. Material and Methods

### 2.1. Extract Preparation and Characterization

Extraction of phenolic compounds: the glycosylated polyphenols are derived from the treatment of waste water in the food industry, in particular those resulting, but not limited to, the production of olive oil (vegetation water or waste water mill). The process, making use of the molecular imprinting technique, consents the selective extraction only of a particular pattern of glycosylated polyphenols (patent appl. under filling).

### 2.2. HPLC Analysis

The HPLC analysis for analytical separations was performed in Shimadzu SCL-10Avp equipped with a Diode Array SPD-M10Avp. A Phenomenex Hydro-RP column (250 × 4,6 mm; 4 *μ*m; 80 A) maintained at 25°C, was used. Elution was performed at a flow rate of 1.0 mL/min, using as the mobile phase a mixture of water/acetic acid (99.95 : 0.05 v/v) (solvent A) and methanol (solvent B). The solvent gradient changed according to the following conditions: from 95% (A) : 5% (B) to 75% (A) : 25% (B) in 10 min, to 60% (A) : 40% (B) in 10 min, to 50% (A) : 50% (B) in 10 min, and to 100% (B) in 10 min; 100% (B) was maintained for 5 min, and the run was ended. Quantification of phenols was carried out at 270 nm. Triplicate determinations were made.

### 2.3. Mass Spectrometry (LC-MS) of the Extract

To confirm the identity of tasted peaks, the extract was analyzed using an HPLC Shimadzu SCL-10Avp equipped with an LCMS-QP8000*α* with an APCI interface. A Phenomenex Hydro-RP column (250 × 4, 6 mm; 4 *μ*m; 80 A) at a flow rate of 1 mL/min was used. The solvent composition and gradient profile were the same as for previous analytical procedure. The ESI mass spectra in the negative ion mode were obtained under the following conditions: capillary temperature, 250°C; lens, skimmer, and octupole voltages were set to get optimal response under these conditions; the spectra show enough ionic fragmentation to verify structural information from the protonated molecular ion. This analysis allowed us to confirm a content >98% of glycosylated polyphenols.

### 2.4. Cell Cultures

IB3-1 cells, derived from a CF patient with a ΔF508/W1282X mutant genotype and immortalized with adeno 12/SV40, were grown in LHC-8 supplemented with 5% FBS in the absence of gentamycin, at 37°C/5% CO_2_ [6]. The effects of active principles were analyzed as elsewhere described.

#### 2.4.1. Proliferation Assay

**Figure 6:**
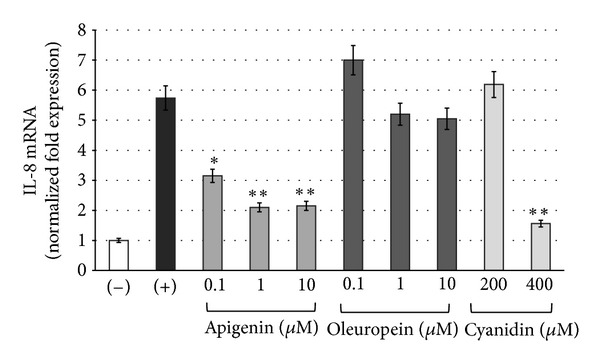
Effects of apigenin, oleuropein, and cyanidin chloride on IL-8 gene expression in TNF-*α*-treated cells. IB3-1 cells were exposed for 24 h to the indicated concentrations of compounds in the presence of TNF-*α* as described in methods. After this incubation time, RNAs and cellular supernatants were isolated. Quantitative RT-PCR was performed, and IL-8 mRNA content was quantified in respect to control uninduced IB3-1 cells (−); (+) = TNF-*α*-treated IB3-1 cells. *P < 0.05;  **P < 0.01.

**Figure 7:**
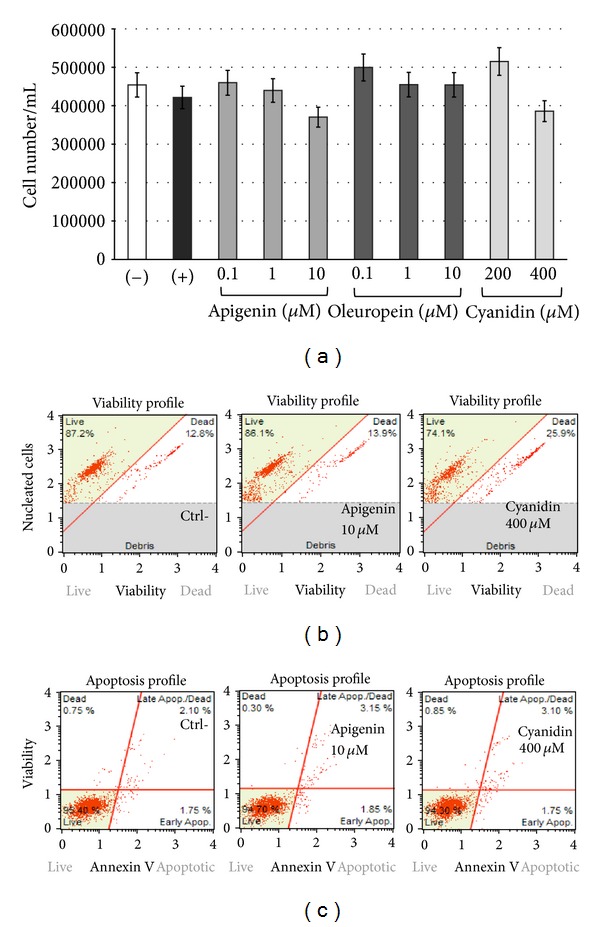
(a) Effects of apigenin, oleuropein, and cyanidin chloride on cell proliferation. (−): untreated IB3-1 cells; (+): TNF-*α*-treated IB3-1 cells (24 h); (b) viability profile of IB3-1 cells treated for 24 h with apigenin 10 *μ*M and cyanidin chlorhide (400 *μ*M); and (c) apoptosis profile of of IB3-1 cells treated for 24 h with apigenin 10 *μ*M and cyanidin chlorhide (400 *μ*M).

Monolayers of 70% confluent IB3-1 cells were seeded in 6-well plates in LHC-8 medium in the presence of 5% FBS. After 24 h, pure derivatives (apigenin, oleuropein, and cyanidin chloride) were added at serial dilutions (as indicate in Figures 6 and 7) before stimulation with TNF-*α* 80 ng/mL (ISOkine, ORF Genetics, Kopavogur, Iceland) and incubated for further 2-3 day. After this time, cells were washed with PBS and detached with trypsin/EDTA. Cells were suspended in physiological solution and counted with a ZBI Coulter Counter (Coulter Electronics, Hialeah, FL, USA). The cell number/mL was determined as IC_50_ after 2 days of culture when untreated cells are in log phase of cell growth.

#### 2.4.2. Viability Assay

Cell counting and viability assay on IB3-1 cells, untreated and treated with increasing doses of either apigenin and cyanidin chloride for 24 h, were performed with automated “Muse” (Merck Millipore, Billerica, MA, USA) method. This method was carried out according to the instructions supplied by the manufacturer, which encompass an in-house method of nuclear staining for the assessment of cell viability. Cells were washed with sterile PBS 1X, trypsinized, suspended, and diluted (1 : 20) with the one step addition of the mix-and-read Muse Count & Viability reagent. Data from prepared samples are acquired and recorded utilizing the Count & Viability Software Module (Merck Millipore, Billerica, MA, USA).

#### 2.4.3. Apoptosis Assay

Annexin V and Dead Cell assay on IB3-1 cells, untreated and treated for 24 h with increasing doses of either apigenin and cyanidin chloride, were performed with “Muse” (Merck Millipore, Billerica, MA, USA) method, according to the instructions supplied by the manufacturer. This procedure utilizes Annexin V to detect phosphatidyl serine (PS) on the external membrane of apoptotic cells. A dead cell marker is also used as an indicator of cell membrane structural integrity. It is excluded from live, healthy cells, as well as early apoptotic cells. Four populations of cells can be distinguished in this assay. Cells were washed with sterile PBS 1X, trypsinized, suspended, and diluted (1 : 2) with the one step addition of the Muse Annexin V & Dead Cell reagent. After incubation of 20 min at room temperature, samples were analyzed, using Triton X 0.01%, as positive control. Data from prepared samples are acquired and recorded utilizing the Annexin V and Dead Cell Software Module (Millipore).

### 2.5. Electrophoretic Mobility Shift Assay (EMSA)

Electrophoretic mobility shift assays were performed by using double-stranded ^32^P-labeled oligonucleotides as target DNA. Binding reactions were set up as described elsewhere [19] in binding buffer (10% glycerol, 0.05% NP-40, 10 mM Tris-HCl pH 7.5, 50 mM NaCl, 0.5 mM DTT, and 10 mM MgCl_2_), in the presence of poly (dI:dC).poly (dI:dC) (Pharmacia, Uppsala, Sweden), 2–5 mg of crude nuclear extracts, or 0.1 mL/20 mL of NF-kappaB-p50 (50 gsu) (PROMEGA, Madison, WI, USA), and 0.25 ng of labeled oligonucleotide, in a total volume of 20 mL (10). After 30 min binding at room temperature, samples were electrophoresed at constant voltage (200 V for 1 h) through low ionic strength (0.25× TBE buffer) (1× TBE/40.089 M Tris-borate, 0.002 M EDTA) on 6% polyacrylamide gels until tracking dye (bromophenol blue) reached the end of a 16 cm slab. Gels were dried and exposed for autoradiography with intensifying screens at −80°C. In these experiments, DNA/protein complexes migrate through the gel with slower efficiency. In studies on the inhibitors of protein/DNA interactions, addition of the reagents was as follows: (i) poly (dI:dC).poly (dI:dC); (ii) labeled oligonucleotides mimicking the binding sites for TF to be modulated; (iii) active principles; (iv) binding buffer; and (v) nuclear factors.

The nucleotide sequences of double-stranded target DNA utilized in these experiments were 5′-CGC TGG GGA CTT TCC ACG G-3′ (sense strand, NF-kappaB). The synthetic oligonucleotides utilized in this study were purchased from Sigma-Genosys (Sigma-Genosys, Cambs, UK). 

### 2.6. Quantification of IL-8 mRNA Content

Total RNA was extracted using TRIzol Reagent (Sigma, St. Louis, MO, USA) following the manufacturer's instructions. Reverse transcription (RT) was performed using Reverse Transcription System kit (Promega, Madison, WI, USA): 1 *μ*g of total RNA was reverse transcribed in the presence of 5 mM MgCl_2_, 1× reverse transcription buffer (10 mM Tris–HCl, 50 mM KCl, 0.1% Triton X-100), 1 mM each dNTPs, 20 U recombinant RNasin Ribonuclease Inhibitor, 15 U AMV reverse transcriptase, 0.5 *μ*g Oligo (dT)_15_ primers in a total volume of 20 *μ*L for 10 min at 70°C and 60 min at 42°C. The resulting cDNA was quantified by relative quantitative real-time PCR (real-time qPCR). For the Real-time qPCR, 1 *μ*L of cDNA was used for each Sybr Green real-time PCR to quantify the relative IL-8 expression. Each 25 *μ*L of total reaction volume contained 1 *μ*L of cDNA, 10 pmol of primers, and 1 × iQ SYBR Green Supermix (Bio-Rad Laboratories Inc., Hercules, CA, USA). Real-time PCRs were performed for a total of 40 cycles (95°C for 10 s, 68°C for 30 s, and 72°C for 60 s) using an iCycler IQ (Bio-Rad Laboratories Inc., Hercules, CA). Primer sequences were IL-8 forward: 5′-GTG CAG TTT TGC CAA GGA GT-3′ and IL-8 reverse: 5′-TTA TGA ATT CTC AGC CCT CTT CAA AAA CTT CTC-3′. Results were collected with Bio-Rad iQ5 software. The relative to quantification of gene expression was performed utilizing the comparative threshold (*C*_*T*_) method. Changes in mRNA expression level were calculated following normalization with the GAPDH calibrator gene and expressed as fold change over untreated samples.

### 2.7. Bio-Plex Analysis

Cytokines in tissue culture supernatants released from the cells under analysis were measured by Bio-Plex cytokine assay (Bio-Rad Laboratories, Hercules, CA, USA) [20, 21] as described by the manufacturer. The Bio-Plex cytokine assay is designed for the multiplexed quantitative measurement of multiple cytokines in a single well using as little as 50 *μ*L of sample. In our experiments, the premixed multiplex beads of the Bio-Plex human cytokines (IL-8, IL-6, IL-1, G-CSF, MPC-1, RANTES, and VEGF) were used. 50 *μ*L of cytokine standards or samples (supernatants recovered from treated cells and diluted to 2 *μ*g/*μ*L) were incubated with 50 *μ*L of anticytokine-conjugated beads in 96-well filter plates for 30 min at room temperature with shaking. Plates were then washed by vacuum filtration three times with 100 *μ*L of Bio-Plex wash buffer, 25 *μ*L of diluted detection antibody were added, and plates were incubated for 30 min at room temperature with shaking. After three filter washes, 50 *μ*L of streptavidin-phycoerythrin was added, and the plates were incubated for 10 min at room temperature with shaking. Finally, plates were washed by vacuum filtration three times, beads were suspended in Bio-Plex assay buffer, and samples were analyzed on a Bio-Rad 96-well plate reader using the Bio-Plex Suspension Array System and Bio-Plex Manager software (Bio-Rad Laboratories, Hercules, CA, USA).

### 2.8. Statistics

Results are expressed as mean ± standard deviation (SD). Comparisons between groups were made by using paired Student's *t*-test and a one-way analysis of variance (ANOVA). Differences were considered significant when *P* < 0.05 and highly significant when *P* < 0.01.

## 3. Results

### 3.1. Extraction of Polyphenolic Mixtures from Olive Oil Water Mill Waste: Analysis of Major Components

The mixture extracted from the olive oil water mill waste contains mainly glycosylated polyphenols. The mixture was analyzed using liquid chromatography (HPLC) followed by mass spectrometry (MS), in order to recognize and quantify the present polyphenols.

This analysis allowed us to confirm a content >98% of glycosylated polyphenols. The molecular fragment more represented in the components, both in free form and as contained within the molecular structure of other polyphenols, is hydroxytyrosol; glycosides containing this structure represent 80% of the components of the mixture. They are oleuropein, verbascoside, salidroside, hydroxy-salidroside and its isomers, luteolin, rutin, apigenin, and anthocyanins. 

The amounts of these compounds were found in the following ranges (patent application): free hydroxytyrosol 0-1%, glycosylated hydroxytyrosol 0–5%, verbascoside, isomers and derivatives that maintain the structure of verbascoside 5–35%, oleuropein isomers and derivatives that retain the structure of oleuropein 30–50%, nuzhenide, oleoside, and other glycosides 5–30%, equivalent of hydroxytyrosol >30%.

In addition, are present, although at lower concentration, other polyphenols, including quercetin, apigenin, cyanidin chloride, p-coumaric acid, caffeic acid, ferulic acid, tyrosol, syringic acid, gallic acid, and rutin hydrate.

Quercetin, apigenin, cyanidin chloride, p-coumaric acid, caffeic acid, ferulic acid, tyrosol, syringic acid, gallic acid, rutin hydrate and oleuropein were found pure (Sigma-Aldrich).

### 3.2. Inhibition of NF-KappaB/DNA Interactions

To analyze in EMSA (Electrophoretic Mobility Shift Assay) studies the biological effects of the natural molecular imprinting olive-extract (NMIO-extract), nuclear extracts of K562 cell line containing NF-kappaB protein were prepared and employed for the binding reactions.

The obtained results, shown in Figure 3, demonstrate that the olive extract is active in inhibiting NF-*κ*B/DNA interactions, even at high concentrations (400 ng/*μ*L).

The same experiment was performed utilizing purified p50 NF-kappaB. In this way, we observed that the NMIO-extract is active at lower concentrations (<0.1 *μ*g/*μ*L), as shown in Figure 4, displaying IC_50_ values between 25 and 50 ng/*μ*L.

Afterwards, in order to understand what components of the NMIO-extract were responsible of the observed activity, we further analyzed selected pure components from the extract (Figure 5). Indeed, among the compounds tested in inhibiting NF-*κ*B/DNA interactions, apigenin, oleuropein, and cyanidin chloride were the most efficient (Figure 5). Our results show that 100 *μ*M of these latter molecules fully suppress the interactions between p50 NF-kappaB and target DNA, while others active principles have demonstrated activity only starting from high concentrations (1–10 mM). In conclusion, this set of experiments demonstrates that these extracts from olives waste water are active in inhibiting the interactions between NF-kappaB and target DNA, and, moreover, that the active components are represented by compounds containing a rather different skeleton (i.e., flavonoid and tyrosol).

### 3.3. Inhibition of IL-8 mRNA Accumulation in TNF-Alpha-Treated IB3-1 Cystic Fibrosis Cells

Starting from the results described above, we have decided to further characterize apigenin, oleuropein, and cyanidine chloride for their potential activities on the expression of interleukin-8 (IL-8) gene. It is firmly established that IL-8 gene expression is regulated by NF-*κ*B transcription factor [11–13, 22]; therefore, since molecules inhibiting NF-*κ*B/DNA interactions might exhibit inhibitory activities on NF-*κ*B-regulated genes [16–18], we were interested to determine the activity of active principles on IL-8 gene expression.

We therefore employed, in our experiments, IB3-1 CF cells, incubated for 5 h in the presence of different concentrations of the selected three compounds and then treated with TNF-*α* (80 ng/mL). After 1 day of incubation, cellular RNA was isolated for Q-RT-PCR analysis. In the IB3-1 CF cellular model, NF-*κ*B dependent genes, including the gene coding for the proinflammatory protein IL-8, generally are activated following infection with *P. aeruginosa*, or treatment with TNF-*α* or IL-1*β*  [7, 22, 23]. This feature is very important in the pathophysiology of CF, since several clinical complications are caused by exacerbation of this inflammatory response. 

The acquired results, resumed and reported in Figure 6, demonstrate that apigenin inhibits TNF-*α*-induced IL-8 mRNA accumulation exhibiting fair dose-response effects, starting from 0.1 *μ*M concentration. Very remarkably apigenin is able to inhibit IL-8 gene expression without major effects on IB3-1 cell growth and viability (data described in Figures 7(a) and 7(b)). Moreover, under the experimental conditions shown in Figures 6 and 7, no induction of apoptosis was found in apigenin-treated cells (Figure 7(c)). These data show that treatment of IB3-1 CF cells with apigenin significantly reduces the TNF-*α*-dependent transcription of the proinflammatory mediator IL-8.

Also cyanidin chloride has been shown to possess biological activity by inhibiting the expression of IL-8 in IB3-1 cells, returning it to basal level when cells were treated with 400 *μ*M cyanidin. Although the concentration at which cyanidin is active seems elevated, we observed no cytotoxic effects on cultured cells (Figure 7).

On the contrary, oleuropein showed no activity even at higher concentrations (up to 400 *μ*M; data not shown).

**Figure 8:**
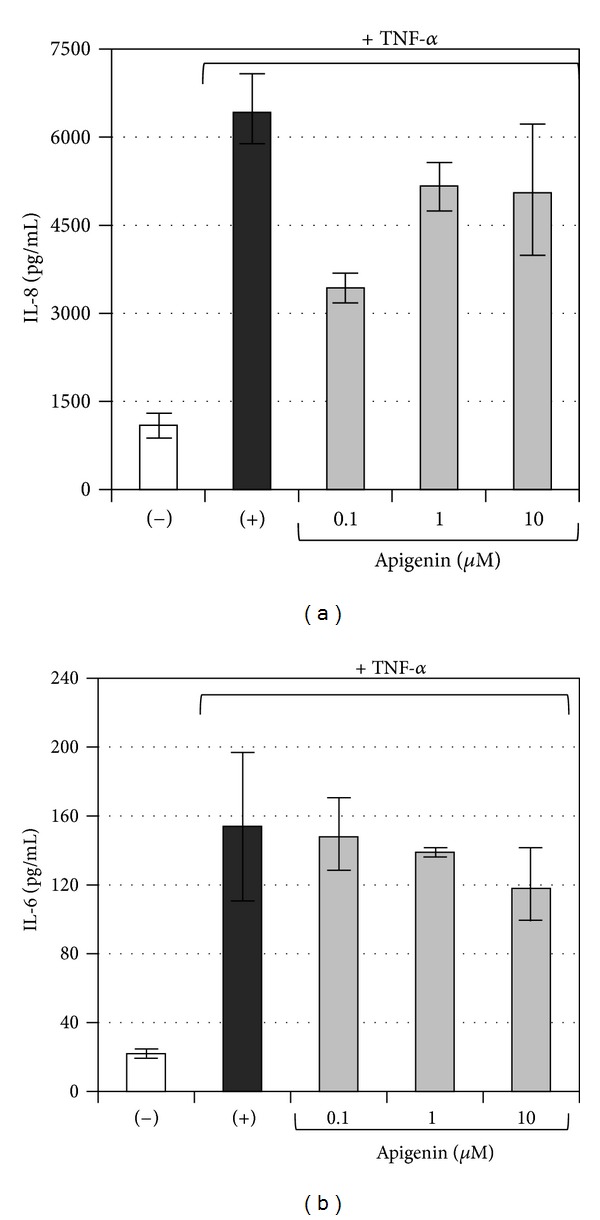
Bio-Plex analysis of IL-8 (a) and IL-6 (b) proteins present in supernatants. (−) untreated IB3-1 cells; (+) IB3-1 cells treated with TNF-*α*.

Our results have been also confirmed using Bio-Plex technology to analyze the release of IL-8 protein (pg/mL) in supernatants recovered from IB3-1 cells induced with TNF-*α* and treated with apigenin, active principle that seems to be the more active derivative. These supernatants were collected before performing the RNA extraction for real-time PCR analysis. The results, shown in Figure 8(a), confirmed the inhibitory activity of apigenin on expression of IL-8 protein in IB3-1 cell model. Also the cytokine IL-6 appears to be modulated by apigenin 10 *μ*M (Figure 8(b)), while, the remaining cytokines (IL-1, G-CSF, MCP-1, RANTES, and VEGF), analyzed with the BioPlex assay, have not demonstrated variations of concentrations (data not shown).

It should be underlined that the differences in the concentrations of apigenin and cyanidin chloride needed to achieve biological effects on NF-*κ*B/DNA interactions in EMSA assays (Figures 5 and 6) and on IL-8 gene expression in the *in vitro* IB3-1 cellular system are not unexpected, since they are very different experimental model systems, as also reported by us in several paper [24–26]. 

## 4. Discussion and Conclusions

In the field of research about cystic fibrosis, the study of novel and innovative drugs for the treatment of this pathology is constantly evolving in order to ameliorate the clinical conditions of patients. There are different classes of commercial and experimental remedies for CF cure, such as antivirulence derivatives, gene correction/potentiator drugs, and anti-inflammatory treatments. In the conventional treatment of CF, the commonly utilized drugs are nonsteroidal anti-inflammatory drugs (NSAIDs) and steroids derivatives.

The search for modern therapies that try to counteract the inflammation in CF patients is aimed at finding new potential anti-inflammatory drugs with different mechanisms of action that may replace the use of certain drugs, such as steroids that possess, in addition to great benefits, many known side effects.

In inflammatory processes that involve patients with CF, but also, for example, in patients with inflammatory bowel diseases (IBDs) and rheumatoid arthritis (RA), NF-kappaB TF plays a crucial role. The activation of the inflammatory response depends on several transcription factors including NF-kappaB, deputy to regulate the activation of many genes that determine the synthesis of cytokines, chemokines, adhesion molecules, and other proteins involved in inflammation, apoptosis, and in the regulation of cell proliferation. NF-kappaB is activated by extracellular stimuli including cytokines, UV rays, free radicals, viral and bacterial products. The NF-kappaB dimers, following a cascade of intracellular biochemical events, are left free to move into the nucleus, where they activate specific proinflammatory genes, inducing transcription of cytokines (TNF-alpha, IL-1, IL-6, IL-8), chemokines (MIP-2, Cinch), adhesion molecules (ICAM-1, E-selectin), and enzymes such as cyclooxygenase-2 and inducible NO synthase (iNOS) (AS Baldwin 2001). For this reason, it is extremely interesting to study new potential anti-inflammatory drugs, which inhibit the action of NF-kappaB and the subsequent production of cytokines (particularly IL-8) and biological factors responsible for the chronic inflammatory state through a mechanism of action alternative to the drugs now utilized in therapy.

We have focused our attention on a new extract derived from olive mill waste water. This strategy is based on the assumption that within by-products of the food and agro-industry, compounds of possible interest in biomedicine, strongly supporting the full usage of industrial materials, including a variety of waste by-products might be present. The most represented active ingredient of the studied mixture, as molecular structure among other polyphenols, is the hydroxytyrosol: glycosides containing this active substance represents 80% of the extract and makes the content of hydroxytyrosol, expressed in equivalents, up to 30%. This glycosides are oleuropein, verbascoside, salidroside, salidroside and its hydroxyl isomers, luteolin, rutin, apigenin and anthocyanins.

The quantities of these compounds in a typical extract are reported below:

(i) hydroxytyrosol free: 1%,
(ii) hydroxytyrosol glycosylated: 5%,
(iii) verbascoside, isomers, and derivatives that retain the structure of verbascoside: 30%,
(iv) oleuropein isomers, and derivatives which retain the structure of oleuropein: 34%,
(v) nuzhenide, oleoside, and other minor glycosides: 30%.

As described in detail, we have confirmed, analyzing all the pure identified derivatives, that two phenolic compounds, apigenin and cyanidin chloride, are extremely interesting in modulating gene expression of IL-8. 

It is well known that apigenin and cyanidin possess anti-inflammatory effect on several biological models. Since 1993, apigenin activity was described starting from the first *in vivo *study of Fuchs and Milbradt [27], demonstrating that liposomal apigenin-7-glucoside was able to inhibit, in a dose-dependent manner, skin inflammation caused by xanthine-oxidase and cumene hydroperoxide in rats. However, the *in vivo* biological activity of cyanidin to prevent inflammation and colon cancer has been described and published, for the first time, in 2001 by Seeram and coworkers [28]. Mainly protocatechuic acid, the predominant degradation product of anthocyanins, demonstrated antioxidant activity comparable to those of different commercial antioxidants.

In the following years, studies on anti-inflammatory activity of these two molecules have continued, leading to the publication of several interesting results.

Some studies have demonstrated the antioxidant effects of cyanidin properties that may protect cells from oxidative damage reducing the risk of cardiovascular diseases and cancer. It seems that cyanidin can inhibit the development of obesity and diabetes, in addition to providing anti-inflammatory effects [29, 30]. Moreover, cyanidin and their metabolites are able to repress the production of pro-inflammatory cytokines such as TNF-*α* and IL-1*β* of inflammatory mediators such as NO, prostaglandin PGE2, the nitric oxide synthase INOS gene, and cyclooxygenase-2. They are also able to inhibit the phosphorylation of IkB-*α* and the nuclear translocation of NF-kappaB [31]. 

It is also known that apigenin possess anti-inflammatory activity, blocking the NF-*κ*B activation pathways in the human mast cell line (HMC-1) [32]. In addition, different studies have been performed also *in vivo* to verify the biological activity of apigenin. Pang and coworkers [33]  demonstrated that apigenin could reduce the airway inflammation and hyper reactivity by down-regulating the expressions of pulmonary GATA-3 and Th2 cytokines in asthmatic mice, while the published data of Li and collaborators [34] clearly showed that apigenin exhibits an anti-inflammatory activity in a murine asthma model. In a second paper, Nicholas et al. demonstrated that apigenin inhibits *in vivo* LPS-induced TNF and the mortality induced by lethal doses of LPS [35]. Taken together, and considering our data on cystic fibrosis cells, apigenin deserves, after optimizing a suitable delivery system, to be analyzed in cystic fibrosis mouse model systems, following the protocol schemes reported in the representative examples regarding studies on the *in vivo* effects of rapamycin, levofloxacin, and azithromycin [36–38].

Moreover, it was demonstrated that apigenin suppresses di-(2-ethylhexyl) phthalate-(DEHP) stimulated expression of intercellular adhesion molecule-1-(ICAM-1) at the mRNA and protein levels [39]. DEHP in house dust seems to be associated with allergic inflammatory symptoms mainly in children. Authors shown that a treatment with apigenin also led to a dose-dependent inhibition of mRNA and protein expression of IL-6 and IL-8 in DEHP-stimulated umbilical vein endothelial cells (HUVECs).

It was also shown that apigenin, such as genistein, kaempferol, and quercetin, seems to play an interesting role in activating cystic fibrosis transmembrane conductance regulator-(CFTR-) mediated Cl currents in human airway epithelium [40]. More recently, Lim and collaborators [41], studying the modulation of ΔF-508 CFTR trafficking and function in IB3-1 human cells, reported the beneficial effects in cystic fibrosis of apigenin, genistein, kaempferol, and quercetin in association with 4-PBA (4-phenylbutyrate). In fact, immunofluorescence assays have demonstrated a favorable change in the intracellular distribution of CFTR with treatments of apigenin and increase in Cl-conductance as measured by Cl-efflux in cells that were treated for 24 h with 4-PBA and 5 microM apigenin.

In this context, we would like to disclose our promising results, concerning principally apigenin and its anti-inflammatory activities, modulating the expression of IL-8 under the control of NF-kappaB in CF IB3-1 cells exposed to TNF-*α*.

Finally, the approach described in the present paper should be considered in the context of research programs based on management of bioproducts of human activity (waste oil) for deriving low-cost product for the health system. To our knowledge, this is the first paper to report that apigenin and cyanidin chloride can be extracted from olive mill waste water and the first report showing that cyanidin chloride retains biological activity on IL-8 expression through the NF-*κ*B pathway.

## References

[1] Welsh MJ, Tsui LC, Boat TF, Beaudet AL, Scriver CR, Beaudet AL, Sly WS, Valle D (1995). Cystic fibrosis. *The Metabolic and Molecular Bases of Inherited Disease*.

[2] Becker MN, Sauer MS, Muhlebach MS (2004). Cytokine secretion by cystic fibrosis airway epithelial cells. *American Journal of Respiratory and Critical Care Medicine*.

[3] Black HR, Yankaskas JR, Johnson LG, Noah TL (1998). Interleukin-8 production by cystic fibrosis nasal epithelial cells after tumor necrosis factor-*α* and respiratory syncytial virus stimulation. *American Journal of Respiratory Cell and Molecular Biology*.

[4] Bonfield TL, Panuska JR, Konstan MW (1995). Inflammatory cytokines in cystic fibrosis lungs. *American Journal of Respiratory and Critical Care Medicine*.

[5] Carpagnano GE, Barnes PJ, Geddes DM, Hodson ME, Kharitonov SA (2003). Increased leukotriene B4 and interleukin-6 in exhaled breath condensate in cystic fibrosis. *American Journal of Respiratory and Critical Care Medicine*.

[6] Tamanini A, Borgatti M, Finotti A (2011). Trimethylangelicin reduces IL-8 transcription and potentiates CFTR function. *American Journal of Physiology*.

[7] Nicolis E, Lampronti I, Dechecchi MC (2009). Modulation of expression of IL-8 gene in bronchial epithelial cells by 5-methoxypsoralen. *International Immunopharmacology*.

[8] Nicolis E, Lampronti I, Dechecchi MC (2008). Pyrogallol, an active compound from the medicinal plant *Emblica officinalis*, regulates expression of pro-inflammatory genes in bronchial epithelial cells. *International Immunopharmacology*.

[9] Taamalli A, Arráez-Román D, Zarrouk M, Valverde J, Segura-Carretero A, Fernández-Gutiérrez A (2012). The occurrence and bioactivity of polyphenols in Tunisian olive products and by-products: a review. *Journal of Food Science*.

[10] Borgatti M, Chilin A, Piccagli L (2011). Development of a novel furocoumarin derivative inhibiting NF-kappaB dependent biological functions: design, synthesis and biological effects. *European Journal of Medicinal Chemistry*.

[11] Luqman S, Pezzuto JM (2010). NF*κ*B: a promising target for natural products in cancer chemoprevention. *Phytotherapy Research*.

[12] Mantovani A, Marchesi F, Porta C, Allavena P, Sica A (2008). Linking inflammation reactions to cancer: novel targets for therapeutic strategies. *Advances in Experimental Medicine and Biology*.

[13] Sethi G, Sung B, Aggarwal BB (2008). Nuclear factor-*κ*B activation: from bench to bedside. *Experimental Biology and Medicine*.

[14] Ramström O, Skudar K, Haines J, Patel P, Brüggemann O (2001). Food analyses using molecularly imprinted polymers. *Journal of Agriculture and Food Chemistry*.

[15] Michailof C, Manesiotis P, Panayiotou C (2008). Synthesis of caffeic acid and p-hydroxybenzoic acid molecularly imprinted polymers and their application for the selective extraction of polyphenols from olive mill waste waters. *Journal of Chromatography A*.

[16] Boucher RC (2004). New concepts of the pathogenesis of cystic fibrosis lung disease. *European Respiratory Journal*.

[17] Khan TZ, Wagener JS, Bost T, Martinez J, Accurso FJ, Riches DWH (1995). Early pulmonary inflammation in infants with cystic fibrosis. *American Journal of Respiratory and Critical Care Medicine*.

[18] Bezzerri V, Borgatti M, Finotti A, Tamanini A, Gambari R, Cabrini G (2011). Mapping the transcriptional machinery of the IL-8 gene in human bronchial epithelial cells. *The Journal of Immunology*.

[19] Lampronti I, Bianchi N, Borgatti M (2005). Effects of vanadium complexes on cell growth of human leukemia cells and protein-DNA interactions. *Oncology Reports*.

[20] Borgatti M, Rizzo R, Dal Canto MB (2008). Release of sICAM-1 in oocytes and in vitro fertilized human embryos. *PLoS ONE*.

[21] Penolazzi L, Lambertini E, Tavanti E (2001). Evaluation of chemokine and cytokine profiles in osteoblast progenitors from umbilical cord blood stem cells by BIO-PLEX technology. *Journal of Clinical Investigation*.

[22] Bezzerri V, Borgatti M, Nicolis E (2008). Transcription factor oligodeoxynucleotides to NF-*κ*B inhibit transcription of IL-8 in bronchial cells. *American Journal of Respiratory Cell and Molecular Biology*.

[23] Gambari Roberto R, Borgatti M, Bezzerri V (2010). Decoy oligodeoxyribonucleotides and peptide nucleic acids-DNA chimeras targeting nuclear factor kappa-B: inhibition of IL-8 gene expression in cystic fibrosis cells infected with *Pseudomonas aeruginosa*. *Biochemical Pharmacology*.

[24] Marzaro G, Guiotto A, Borgatti M (2013). Psoralen derivatives as inhibitors of nf-kappab/dna interaction: synthesis, molecular modeling, 3d-qsar, and biological evaluation. *Journal of Medicinal Chemistry*.

[25] Gambari R, Borgatti M, Lampronti I (2012). Corilagin is a potent inhibitor of NF-kappaB activity and downregulates TNF-alpha induced expression of IL-8 gene in cystic fibrosis IB3-1 cells. *International Immunopharmacology*.

[26] Borgatti M, Chilin A, Piccagli L (2011). Development of a novel furocoumarin derivative inhibiting NF-kappaB dependent biological functions: design, synthesis and biological effects. *European Journal of Medicinal Chemistry*.

[27] Fuchs J, Milbradt R (1993). Skin anti-inflammatory activity of apigenin-7-glucoside in rats. *Arzneimittel-Forschung*.

[28] Seeram NP, Bourquin LD, Nair MG (2001). Degradation products of cyanidin glycosides from tart cherries and their bioactivities. *Journal of Agricultural and Food Chemistry*.

[29] Sasaki R, Nishimura N, Hoshino H (2007). Cyanidin 3-glucoside ameliorates hyperglycemia and insulin sensitivity due to downregulation of retinol binding protein 4 expression in diabetic mice. *Biochemical Pharmacology*.

[30] Muñoz-Espada AC, Watkins BA (2006). Cyanidin attenuates PGE2 production and cyclooxygenase-2 expression in LNCaP human prostate cancer cells. *Journal of Nutritional Biochemistry*.

[31] Min SW, Ryu SN, Kim DH (2010). Anti-inflammatory effects of black rice, cyanidin-3-O-*β*-d-glycoside, and its metabolites, cyanidin and protocatechuic acid. *International Immunopharmacology*.

[32] Kang OH, Lee JH, Kwon DY (2011). Apigenin inhibits release of inflammatory mediators by blocking the NF-*κ*B activation pathways in the HMC-1 cells. *Immunopharmacology and Immunotoxicology*.

[33] Pang LL, Li RR, Zhou LF (2010). Inhibitory effects of apigenin on the expression of GATA-3 and Th2 cytokines in asthmatic mice. *Zhongguo Zhong Xi Yi Jie He Za Zhi*.

[34] Li RR, Pang LL, Du Q, Shi Y, Dai WJ, Yin KS (2010). Apigenin inhibits allergen-induced airway inflammation and switches immune response in a murine model of asthma. *Immunopharmacology and Immunotoxicology*.

[35] Nicholas C, Batra S, Vargo MA (2007). Apigenin blocks lipopolysaccharide-induced lethality in vivo and proinflammatory cytokines expression by inactivating NF-*κ*B through the suppression of p65 phosphorylation. *Journal of Immunology*.

[36] Abdulrahman BA, Khweek AA, Akhter A (2011). Autophagy stimulation by rapamycin suppresses lung inflammation and infection by Burkholderia cenocepacia in a model of cystic fibrosis. *Autophagy*.

[37] Sabet M, Miller CE, Nolan TG, Senekeo-Effenberger K, Dudley MN, Griffith DC (2009). Efficacy of aerosol MP-376, a levofloxacin inhalation solution, in models of mouse lung infection due to *Pseudomonas aeruginosa*. *Antimicrobial Agents and Chemotherapy*.

[38] Hoffmann N, Lee B, Hentzer M (2007). Azithromycin blocks quorum sensing and alginate polymer formation and increases the sensitivity to serum and stationary-growth-phase killing of *Pseudomonas aeruginosa* and attenuates chronic *P. aeruginosa* lung infection in Cftr(-/-) mice. *Antimicrobial Agents and Chemotherapy*.

[39] Wang J, Liao Y, Fan J, Ye T, Sun X, Dong S (2012). Apigenin inhibits the expression of IL-6, IL-8, and ICAM-1 in DEHP-stimulated human umbilical vein endothelial cells and in vivo. *Inflammation*.

[40] Illek B, Fischer H (1998). Flavonoids stimulate Cl conductance of human airway epithelium in vitro and in vivo. *American Journal of Physiology*.

[41] Lim M, McKenzie K, Floyd AD, Kwon E, Zeitlin PL (2004). Modulation of ΔF508 cystic fibrosis transmembrane regulator trafficking and function with 4-phenylbutyrate and flavonoids. *American Journal of Respiratory Cell and Molecular Biology*.

